# Research advances in gestational, neonatal diabetes mellitus and metabolic disorders

**DOI:** 10.3389/fendo.2022.969952

**Published:** 2022-07-29

**Authors:** Ihtisham Bukhari, Furhan Iqbal, Rick Francis Thorne

**Affiliations:** ^1^Henan Provincial and Zhengzhou City Key Laboratory of Non-Coding RNA and Cancer Metabolism, Henan International Join Laboratory of Non-Coding RNA and Metabolism in Cancer, Translational Research Institute of Henan Provincial People’s Hospital, Academy of Medical Sciences, Zhengzhou University, Zhengzhou, China; ^2^Henan Key Laboratory of Helicobacter pylori, Microbiota and Gastrointestinal Cancer, Marshall Medical Research Center, Fifth Affiliated Hospital of Zhengzhou University, Zhengzhou, China; ^3^Zoology Division, Institute of Pure and Applied Biology, Bahauddin Zakariya University, Multan, Pakistan; ^4^School of Environmental and Life Sciences, University of Newcastle, Callaghan, NSW, Australia

**Keywords:** Gestational diabetes mellitus, Neonatal diabetes mellitus, metabolic disorders, Metabolic syndome, diabetes

Diabetes mellitus (DM) is among the most common disorders affecting people of all ages across the globe from neonates to seniors (1). Indeed, DM has emerged as one of the major health concerns of this century with a huge economic burden that continues to grow, especially in low and middle-income counties (2). In addition to type 1 and II diabetes (T2DM), gestational diabetes mellitus (GDM) (3) and neonatal diabetes mellitus (NDM) are now more frequently reported (4). GDM poses significant dangers not only for the mother but also for the developing fetus (5). Unlike the common forms of diabetes, the causes and associated complications of GDM and NDM are not yet completely understood. However, diabetes may also be associated with metabolic syndromes (MetS), a medical term used for a combination of diabetes, high blood pressure (hypertension), and obesity (6). Diabetes also creates a greater risk for coronary heart disease, stroke, and other conditions affecting blood vessels, and notably in pregnancy, a relationship has been established between maternal MetS, GDM, and pregnancy outcomes (7). The current Research Topic aimed to collect studies reporting advancements in clinical and basic research related to GDM, NDM, and associated metabolic disorders. After a rigorous process of selection and review, the current volume presents an authoritative collection of 20 research articles exploring new dimensions in this topic.

Firstly, introducing major players in metabolic disorders, Zhen et al. reviewed the role of kynurenic acid (KYNA) in the pathogenesis and development of several human endocrine and metabolic diseases. KYNA is a signaling molecule and a major player in metabolic diseases inclusive of diabetes and obesity. Their study promotes the notion that KYNA enhances energy metabolism and prevents obesity while reducing insulin resistance and inflammation. And on this basis, the authors suggest KYNA as a potential therapeutic marker/target for metabolic diseases. Alzaim et al. studied the possible association of the vitamin D receptor (VDR) polymorphism (Folk1) with the metabolic syndrome in pregnant women of Arabian descent. Interestingly, they did not find a significant association of metabolic syndrome with Folk1 in VDR; however, carriers of the ff allele were at risk for full maternal MetS, and their study further suggested that the ff genotype in Folk1 could be used as a genetic marker for maternal MetS in pregnant Arab women.

A well-established fact is that type-2 diabetes is abruptly increasing among at-risk populations due to increasingly unhealthy lifestyles among other associated factors (8). Jia et al. studied the involvement of gut microbiota in the regulation of central obesity and type-2 diabetes in Chinese individuals. Notably, they observed a significantly higher percentage of sugar and amino acid metabolism-related gut microbiota in obese patients with T2DM. This suggests that the gut microbiota should be taken into consideration for planning appropriate therapeutic strategies.

Diabetic patients are also prone to develop other morbidities such as liver and kidney dysfunction and lung injury (9). Ding et al. describe a traditional Chinese medicine Jiangtang Tongmai Prescription (JTTMP) that activates SnoN and TGF-β1/Smads signaling pathways to promote diabetic lung injury repair. It is also common that diabetic patients to suffer from a variety of psychological problems. Therefore, considering the psychological care of diabetes patients represents an integral factor for maintenance and recovery regimens. Li et al. studied the effects of individualized nursing and health education (INHE) on patients with T2DM and hypertension. They reported that INHE effectively improves the psychological cognition of T2DM plus hypertension patients, helping to improve blood pressure and blood sugar control.

Raza et al. report the differential expression and association of genes including DMBX1, TAL1, ZFP161, NFIC, NR1H4, SRR, NFKB1, and PDE4B with the pathophysiological development of metabolic disorders including diabetes. Diabetes can affect people of all ages and risk factors may vary with the age group involved (1). Neonatal diabetes is an extremely rare disorder caused by genetic mutations in specific genes including dominantly acting insulin (INS) (10). Ngoc et al. performed a genetic analysis on 70 Vietnamese NDM cases from 2008 to 2021 and report causative mutations in genes known to be associated with NDM. In particular, 55 of the 70 infants (78.5%) harbored mutations in the disease-causing genes ABCC8 and KCNJ1 while 10 subjects showed six distinct heterozygous INS mutations, indicating that INS mutations are the third most common cause of NDM in Vietnam.

Gestational diabetes mellitus can significantly affect the course of pregnancy and its early diagnosis and care improve maternal health and pregnancy outcomes (11). Liu et al. presented a nested case-control study, in which they evaluated serum levels of putrescine in 47 women with GDM, specifically during weeks 8-12 of gestation. Putrescine serum levels were significantly higher in these women during their first trimester, indicating that putrescine can be used as a GDM marker during the first trimester. Adding to the early detection of GDM diagnosis, Zhang et al. compared ankle blood pressure with blood glucose in 179 (52% White European, 48% Asian) pregnant women at 24-28 weeks of gestation. Besides reporting ethnic differences, they suggest that higher ankle blood pressure could be associated with the risk of developing gestational diabetes. Serum uric acid level is a known potential risk factor for GDM and it is also known that hypertension produces adverse birth outcomes. Riis et al. examined the diurnal salivary uric acid (sUA) levels in 44 healthy women during early-mid and late pregnancy. According to their observations, sUA showed an association with maternal pre-pregnancy BMI, age, prior-night sleep duration, and fetal sex. Maternal blood pressure and gestational weight gain also showed significant associations with sUA levels across pregnancy.

Xu et al. enrolled 610 Han Chinese pregnant women to study the short- and long-term adverse fetal and maternal outcomes of hyperglycaemia in pregnancy (HIP). Multivariate logistic regression analysis revealed that previous gestational diabetes mellitus (GDM), pre-pregnancy body mass index (BMI) (≥23 kg/m^2^), and maternal age (≥35 years) were risk factors for HIP in early pregnancy. Further regarding GDM diagnosis, Raets et al. performed glucose challenge tests (GCT) and oral glucose tolerance tests (OGTT) in 1804 pregnant women during weeks 24-28 of gestation. They advised a one-step screening strategy with an OGTT for women at higher risk for GDM, while women without these risk factors were suggested to have a two-step screening strategy with GCT. Mamun and Khan reviewed the available literature regarding COVID-19 infection in pregnant women and concluded that patients with GDM are more vulnerable to coronavirus infection specifically to the COVID-19 Delta Variant of Concern (VOC).

Juchnicka et al. analyzed the differential expression of circulating miRNAs in GDM women and reported that four miRNAs (miR-16-5p, miR-142-3p, miR-144-3p, and miR-320e) showed prominent expression in GDM. Chen et al. shed light on the differential expression and association of circRNAs in GDM patients, reporting the overall top 4 genes (CBLB, ITPR3, NFKBIA, and ICAM1) and corresponding circRNAs (circ-CBLB, circ-ITPR3, circ-NFKBIA, and circ-ICAM1) were related to T cell receptor signaling pathways. This introduces a novel concept where the upregulation of T cell receptor signaling may be involved in GDM development. Moreover, this suggests that monitoring T cell receptor signaling activation during early gestation could represent a novel diagnostic approach. Wang et al. performed GDM risk association studies for rs7747752 in CDKAL1 and GUDCA/DCA polymorphisms and found that patients carrying the rs7747752 allele with low GUDCA/DCA have a significant risk of developing GDM.

While GDM definitively influences pregnancy outcomes, whether GDM has any relation with NDM, or postnatal diabetes was unknown. Jiang et al. created a GDM mouse model to investigate whether GDM causes diabetes in offspring. They concluded that the intrauterine hyperinsulinemia induced hepatic FoxO1 levels, subsequently increasing the expression of the epigenetic writer-reader DNMT3A resulting in differentially methylated regions in IGF2/H19. Their work pointed to a potential molecular mechanism underlying glucose intolerance and insulin resistance in the first male generation of GDM mice but whether GDM contributes to NDM remains unsolved.

## Conclusions and perspectives

This volume contributed by twenty research groups with often different interests highlights many important aspects of diabetes/gestational diabetes. Different biomarkers for diagnosis and prognosis along with potential therapeutic targets were identified along with adding a variety of new information to the topic. Along with the potential diagnostic and therapeutic approaches for gestational diabetes, this includes the involvement of gut microbiota in diabetes along with genetic biomarkers for gestational diabetes and neonatal diabetes. Nevertheless, a major question regarding pregnancy outcomes especially GDM triggers NDM or postnatal diabetes remains unanswered. Together with increasing our biological understanding of different aspects of diabetes, it can be anticipated that these contributions will find broad applications, ranging from purely scientific endeavours to use as clinical guidelines for the treatment of diabetic patients.

## Author contributions

All authors participated equally to this work. All authors contributed to the article and approved the submitted version.

## Conflict of interest

The authors declare that the research was conducted in the absence of any commercial or financial relationships that could be construed as a potential conflict of interest.

## Publisher’s note

All claims expressed in this article are solely those of the authors and do not necessarily represent those of their affiliated organizations, or those of the publisher, the editors and the reviewers. Any product that may be evaluated in this article, or claim that may be made by its manufacturer, is not guaranteed or endorsed by the publisher.
